# Temperature and humidity independently influence thermoregulatory responses during Finnish sauna bathing

**DOI:** 10.1080/23328940.2026.2698162

**Published:** 2026-07-11

**Authors:** Iida Laatikainen-Raussi, Tom Mikkola, Johanna K. Ihalainen, Essi Ahokas

**Affiliations:** a Sport and Health Sciences, University of Jyväskylä, Jyväskylä, Finland; b Finnish Institute of High Performance Sport KIHU, Jyväskylä, Finland

**Keywords:** Heat-therapy, thermal strain, condensation, heat transfer, Finnish sauna

## Abstract

This study examined the effects of sauna air temperature and relative humidity (RH) on acute cardiovascular and thermoregulatory responses under typical Finnish sauna conditions, where participants were free to throw water onto the heated stones (*löyly*). Fifty recreationally active healthy adults (32 males, 18 females, 35.4 ± 10.4 years, 174 ± 9.9 cm, 77 ± 17 kg) completed four 10-minute sauna sessions, each separated by 30-minute recovery. Core temperature (T_core_), skin temperature, heart rate (HR), body mass, and perceptual responses were measured, alongside detailed multi-point environmental monitoring of temperature and relative humidity (RH) monitoring. Generalized estimating equations assessed associations between environmental factors and physiological responses. HR increased markedly during sauna exposure (mean +41.6 bpm), while T_core_ rose moderately (+0.4°C). Temperature and RH were independently associated with increases in HR (β = 1.40 bpm/°C; β = 0.37 bpm/%RH) and T_core_ (β = 0.032°C/°C; β = 0.0079°C/%RH). These associations remained significant after adjustment for sex, physical activity, body fat percentage, and skin surface area. The difference between dew point and skin temperature, used as an index of condensation potential, showed weak associations with HR and no significant relationship with T_core_. Repeated sauna sessions did not meaningfully influence HR responses, and only minor differences were observed in T_core_ between sessions. Perceptual responses were primarily driven by temperature, with smaller contributions from humidity. These findings demonstrate that humidity, beyond temperature, appears to independently contribute to physiological strain during sauna exposure. The results highlight the importance of characterizing environmental conditions and suggest that humidity should be considered when optimizing sauna use for potential health benefits.

## Introduction

Finnish sauna bathing is a form of acute passive heat exposure, characterized by high air temperatures (70–100°C) and typically low to moderate humidity, although short-term humidity can vary substantially depending on steam generation during use. Finnish sauna sessions usually last 5–20 minutes [1,2] and induce rapid, coordinated physiological responses, particularly in the cardiovascular and thermoregulatory systems [3,4]. In the longer-term perspective, heat exposure, especially sauna bathing, appears to be associated with numerous positive health effects [2].

Acute sauna exposure produces cardiovascular responses similar to those of moderate-intensity exercise, such as increased heart rate [5], peripheral vasodilation [2], and changes in plasma volume [6]. It also causes temporary changes in blood pressure and other hemodynamic measures, with effects varying according to heat level and individual factors [5,7–10].

The magnitude of these physiological responses is influenced not only by individual factors, such as fitness level and body composition, but also by the physical characteristics of the sauna environment. Heat transfer occurs primarily through convection from hot air to the skin, while increases in humidity, such as when water is thrown onto the heated stones, enhance heat transfer via steam condensation and the release of latent heat [11,12]. Both temperature and humidity contribute to the overall thermal load and may substantially modify physiological responses. For example, it has been observed that higher relative humidity during exercise increases both core body temperature and heart rate at a constant ambient temperature [13]. Under humid conditions, when skin temperature approaches or falls below the dew point (the temperature at which water vapor condenses into liquid water), water vapor may condense on the skin surface, releasing latent heat and thereby potentially increasing heat transfer to the body. In addition, this condensation reduces the effectiveness of evaporative cooling, potentially increasing thermal strain and cardiovascular responses.

Despite increasing evidence on the acute effects of sauna bathing, the independent and combined effects of sauna air temperature and humidity under real sauna conditions remain poorly understood. In particular, humidity is often inadequately characterized, despite its potential to substantially modify heat transfer and thermoregulatory strain. This limits the ability to optimize sauna use for health and performance.

The aim of the present study was therefore to investigate how sauna air temperature and relative humidity influence acute cardiovascular and thermoregulatory responses during sauna exposure. We hypothesized that (i) both temperature and humidity would independently increase heart rate and core temperature, (ii) humidity would contribute to physiological strain by impairing evaporative cooling, and (iii) indices reflecting condensation potential (i.e. the difference between dew point and skin temperature) would be associated with cardiovascular responses due to enhanced heat transfer to the skin via steam condensation and latent heat release.

## Methods

### Participants

Prior to inclusion in the study, participants were informed of the study purpose and testing procedures. Written informed consent was obtained from all participants. Participants also completed a health screening questionnaire including questions about their usual sauna exposure frequency and their physical activity on a scale 0–7 [14], with a higher score indicating greater levels of physical activity. Exclusion criteria included pregnancy, unstable angina pectoris, recent myocardial infarction, severe aortic stenosis, and acute illness (such as respiratory infections). The study was conducted in accordance with the Declaration of Helsinki [15], except for pre-registration in a database, and ethical approval was provided by the Ethics Committee of the University of Jyväskylä (544/13.00.04.00/2025).

All together 69 healthy adults (23 females, 46 males) participated in the study. Every participant successfully completed the entire protocol, but a total of 19 participants (6 females, 13 males) were excluded due to missing or inaccurate core temperature, skin temperature and/or heart rate data (core temperature *n* = 14, skin temperature *n* = 3, heart rate *n* = 2). The descriptive characteristics of the excluded participants did not differ significantly in height, age, percent of body fat, physical activity, sex or sauna use from the remaining sample. Body mass was significantly higher in the excluded groups (weight: U628.5, *p* = 0.039). The characteristics of those removed from the analysis are compiled in supplementary table 2. A total of 50 participants (32 males, 18 females) were included in the analysis. Background information on the participants can be found in Table 1.

**Table 1.** Participant characteristics, mean (SD). Physical activity based on participants’ questionnaire [14] responses. *PBF = percent of body fat.

	N	Physical activity (0–7)	Age (years)	Height (cm)	Weight (kg)	PBF* (%)
All	50	4.7 (1.9)	35.4 (10.4)	174 (9.9)	77 (17)	24 (7.8)
Female	18	5.4 (1.9)	34 (9.3)	166 (6.5)	63 (8.9)	26 (6.3)
Male	32	4.2 (1.9)	36.3 (11.0)	178 (8.5)	85 (16)	22 (4.6)

90% of the participants were regular sauna bathers (1–3 times per month or more often), 8% reported going to sauna a few times a year, and only one participant reported that they do not normally use a sauna. Thus, the majority can be considered relatively active sauna users. All participants were instructed not to consume any alcohol, and to refrain from additional exercise and heat exposures for 24 hours prior to the experimental trials and on the day of the trials.

### Study design

**Figure 1.**
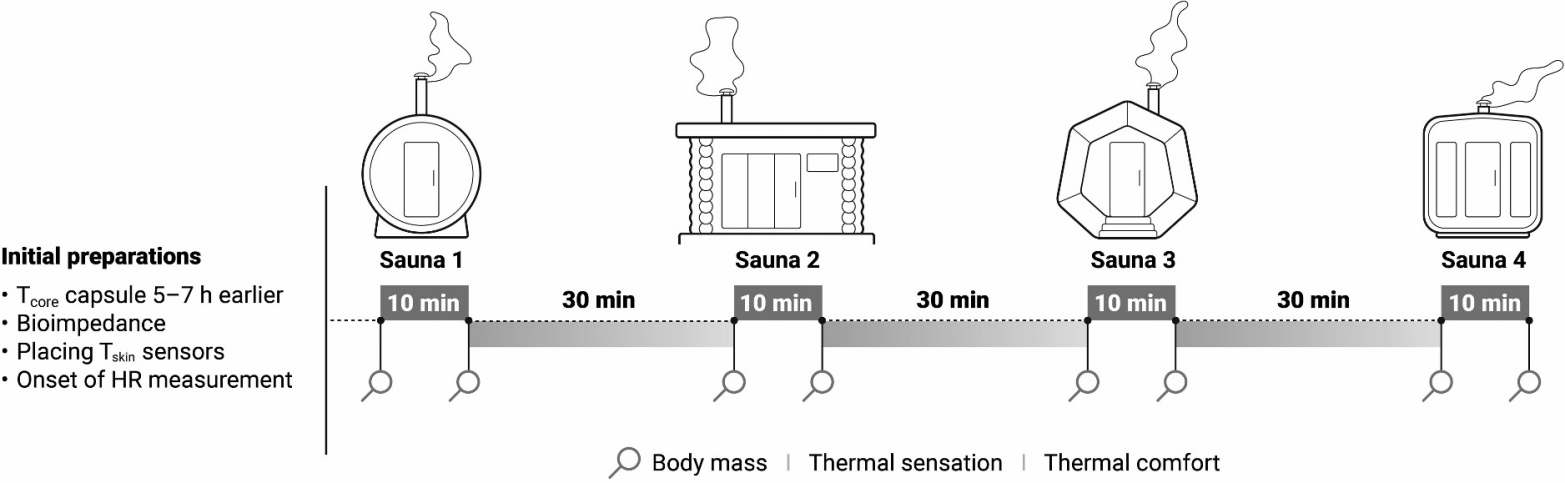
Study design.

Participants completed four separate 10‑minute sauna sessions, each separated by a 30‑minute recovery period at normal room temperature (see Figure 1 for the study design). A typical sauna session lasts 5–20 minutes depending on individual preferences [1,2]. Before the first sauna session participants body composition was measured and skin temperature sensors and a heart‑rate monitoring belt were attached. On the previous day, each participant was provided with an ingestible core temperature capsule, which they were instructed to swallow 5–7 hours before the start of the measurement protocol.

Participants attended the sauna wearing their own swimsuits, in groups of three (*n* = 63) or two (*n* = 6). Preparations and measurements took place in a hall at room temperature (~21.5 °C). Participants were free to drink and eat snacks between the sauna sessions, but not between the pre- and post- sauna weighing.

All four saunas had different heaters (electric, gas, hydrogen, and wood burning). During the first two minutes of each sauna session, participants were not allowed to throw water on the stones of the heater to stabilize the sauna environment. During minutes 2–10 the participants were allowed to throw water freely on the stones. The amount of the water thrown on the heated stones, and in some extend the type of the heater, influenced the relative humidity of the sauna – differently across the groups of three (or two) people.

## Measurements

### Core and skin temperature

T_core_ was recorded using the electronic (gastro-intestinal) temperature pill at 30-s intervals with a precision of 0.1°C (eCelsius; BodyCap, Caen, France.). The pill was ingested by the participant 5–7 h before the start of the sauna bathing. The change in core temperature, ∆T_core_, was calculated as the difference between T_core_ during minutes 0–2 in the sauna and the maximum two-minute moving average during sauna session and the following 30-minute rest period.

T_skin_ was measured at 10-s intervals with three iButtons (DS1922L Thermochron, Wilmington, USA) placed on the forehead, chest, and foot [16], all placed on the right side of the body. Skin temperature was used to calculate the difference between dew point, calculated using the formula by Alduchov & Eskridge [17], and skin temperature (dp-T_skin_). Skin temperature was calculated as a weighted average: 0.4 ×T_skin(forehead)_ +0.4 ×T_skin(chest)_ +0.2 ×T_skin(foot)_ [16].

### Heart rate

Heart rate was measured using a wearable Polar H10 sensor (Polar Electro Oy, Kempele, Finland) throughout the measurement session. Change in heart rate, ∆HR, during every sauna session was calculated as the difference between the highest two-minute average heart rate and the one-minute average heart rate during second minute in the sauna.

### Body composition, mass and sweat loss

Prior to the first sauna exposure, participants’ body composition was assessed using a bioimpedance analyzer (Inbody 720, Biospace Co., Seoul, Korea). Body mass was measured (Kern DE 150K2DL, KERN & SOHN GmbH, Balingen, Germany) prior to and immediately after every sauna session after skin was dried with a towel. Sweat loss was indirectly estimated based on the changes in body mass (m_post sauna_ – m_pre sauna_).

### Perceptual measures

Thermal perception was assessed immediately before and after each sauna session. Participants rated thermal sensation using a 7-point scale (modified from [18]) and thermal comfort using a 4-point scale [19]. Intermediate (0.5) increments were incorporated to enhance resolution.

### Measurements of environmental conditions in the sauna

The temperature and humidity of the sauna room were measured using six thermocouple cords (K type) and six analogue humidity sensors (Vaisala Humidity Module HMM100, Vaisala Ltd, Helsinki, Finland) placed at two different heights (1.73 m from the floor, i.e. typical head level and 0.90 m from the floor, i.e. sitting level) and three different horizontal positions (middle of the sauna room, right side and left side). Data were collected with a DAQ970A data acquisition system (Keysight Technologies, Santa Rosa, USA). Temperature (T) and relative humidity (RH) were calculated as averages from the six different sensors during 2–10 minutes of each sauna session.

### Data and statistical analyses

Data were analyzed using Python (version 3.13.7) with the statsmodels package (version 0.14.6). Data are presented as mean ± standard deviation unless otherwise stated. Statistical significance was set at *p* < 0.05. The effects of sauna room temperature, relative humidity and sauna session order on ∆HR, ∆T_core_, ∆body mass and the perceptual measures (thermal sensation and comfort) were analyzed with a generalized estimating equations model (GEE). In addition, the effects of the difference between dew point and skin temperature, dp-T_skin,_ and sauna session order on ∆HR, ∆T_core_, ∆body mass and the perceptual measures were analyzed with a separate GEE model. Sex, physical activity, body fat percentage and skin surface area were added to these GEE models to assess their potential effect on the changes.

Data were modeled using a Gaussian distribution with an identity link function and participant-level clustering. Given the relatively high resolution of categorical perceptual scales (thermal sensation and comfort) and their linear nature, as well as to facilitate the interpretation of the results, these variables were treated as approximately continuous. Among the predictor variables, sauna session order was treated as a categorical variable. An exchangeable working correlation structure was used to account for within-participant correlations, assuming that repeated observations from the same participant were similarly correlated regardless of sauna session order. Robust (sandwich) standard errors were used to provide valid statistical inference even if the working correlation structure was mis specified. Spearman’s rank correlation coefficients (ρ) were used to assess associations between selected variables due to non-normal distributions and the exploratory nature of these analyses.

Possible differences between included and excluded groups were tested with Mann-Whitney U-test (height, body mass, age, PBF and physical activity), and Chi-Square test was used to test differences in sauna use and sex.

## Results

The mean sauna temperature during minutes 2–10 across all sauna sessions, considering all six measurement points, was 59.9°C (±5.8°C) and the mean relative humidity 40.8% (±15.4%). At the sitting height mean T was 50.8°C (±5.1°C) and RH 53.8% (±20.1%), and at 1.73 meters from the floor mean T 69.0°C (±5.5°C), mean RH 27.7% (±11.7%).

**Figure 2.**
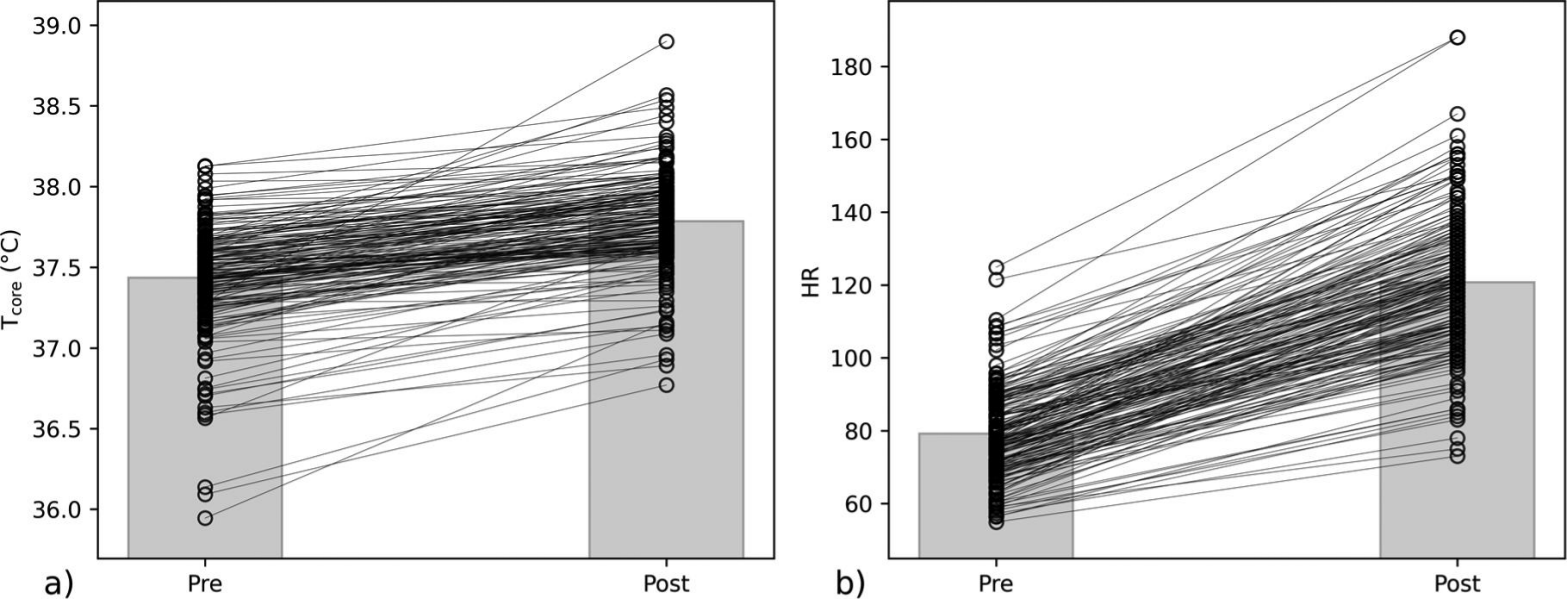
Core temperature (a) and heart rate (b) responses during the sauna session. Pre-values represent baseline measurements at the beginning of the sauna, and post-values represent peak responses calculated as 2-min moving averages during or after the session.

T_core_ and HR values are presented in Figure 2 for every sauna session. T_core_ increased from 37.4 ± 0.3°C to 37.8 ± 0.3°C (Figure 2a) and heart rate increased from 79.2 ± 11.9 bpm to 120.8 ± 18.4 bpm (Figure 2b).

### Heart rate

Overall, temperature and humidity were the only consistent predictors of ΔHR. In a GEE model including sauna session, sauna temperature and relative humidity, T and RH were positively associated with ∆HR (β = 1.42 bpm, 95% CI: 1.09–1.76, *p* = 0.000 and β = 0.38, 95% CI: 0.27–0.48, *p* = 0.000). In a GEE model with sex, physical activity, body fat percentage and skin area added, temperature and humidity remained the only variables with a statistically significant association with ∆HR (β = 1.40 bpm, 95% CI: 1.06–1.73, *p* = 0.000 and β = 0.37, 95% CI: 0.36–0.50, *p* = 0.000). Sauna session order was not significantly associated with changes in ∆HR (supplementary table 1).

In addition, the calculated difference between dew point and skin temperature was positively associated with ∆HR (β = 0.25 bpm, 95% CI: 0.044–0.46, *p* = 0.0176) in a model with sauna sessions, which did not have a statistically significant effect on the change. When sex, physical activity, body fat percentage and skin area were added to the model, no variables remained statistically significant predictors of ∆HR.

**Figure 3.**
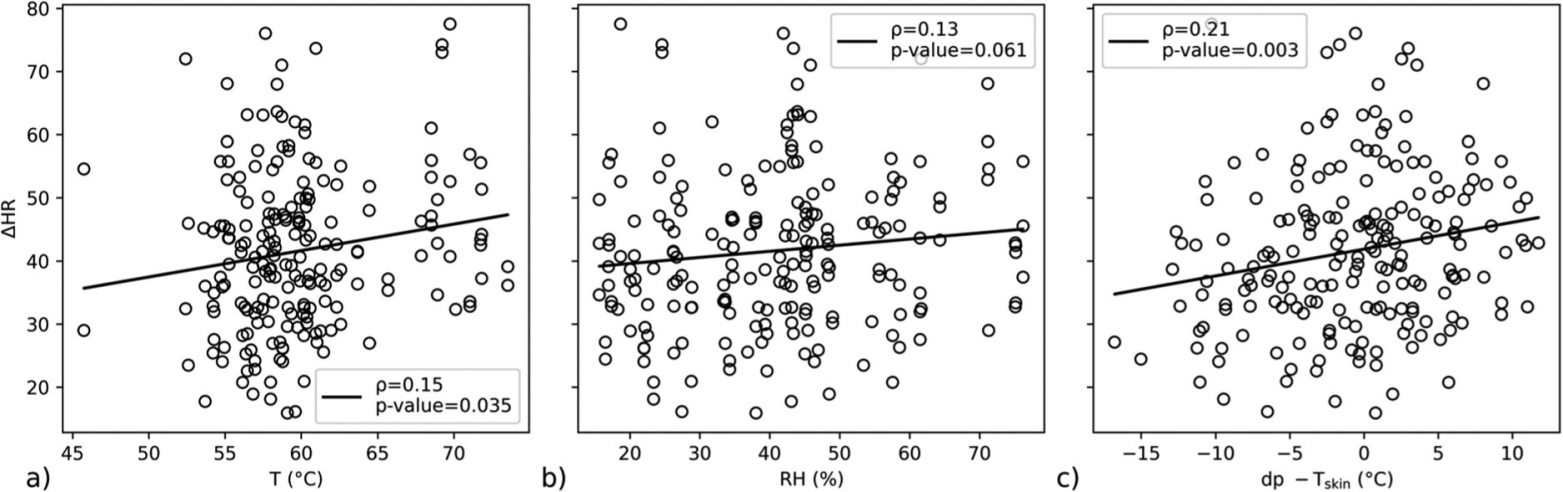
Correlation between T (a), RH (b), or dp-T_skin_ (c) and ∆HR: scatter plots with Spearman’s rho (ρ) and *p*-value.

Figure 3 illustrates the correlations between T, RH, or dp‑T_skin_, and ∆HR, which were weak but statistically significant for T and dp‑T_skin_.

### Core temperature

The results for ∆T_core_ were generally consistent with those for ∆HR. Sauna temperature and relative humidity were positively associated with the change in core temperature (β = 0.032°C, 95% CI: 0.021–0.043, *p* = 0.000 and β = 0.0076°C, 95% CI: 0.0047–0.011, *p* = 0.000), but there was also a statistically significant negative association between ∆T_core_ and third sauna session (β = −0.067°C, 95% CI: −0.20–0.061, *p* = 0.0003). When the model was adjusted also for sex, physical activity, body fat percentage and skin area, T and RH were still positively associated with ∆T_core_ (β = 0.032°C, 95% CI: 0.021–0.043, *p* = 0.000 and β = 0.0079°C, 95% CI: 0.0048–0.011, *p* = 0.000) and physical activity and body fat percentage showed negative associations (β = −0.035°C, *p* = 0.001, 95% CI: −0.056 to −0.014 and β = −0.0041°C, *p* = 0.041, 95% CI: −0.0081 to −0.0002).

The difference between dew point and skin temperature did not show a statistically significant association with ∆T_core_ (β = 0.0052°C, 95% CI: −0.0005–0.011, *p* = 0.071) and nor did the sauna sessions. When sex, physical activity, body fat percentage and skin area were included in the model, only physical activity was negatively associated with the ∆T_core_ (β = −0.040°C, 95% CI: −0.063 to −0.017, *p* = 0.0008).

**Figure 4.**
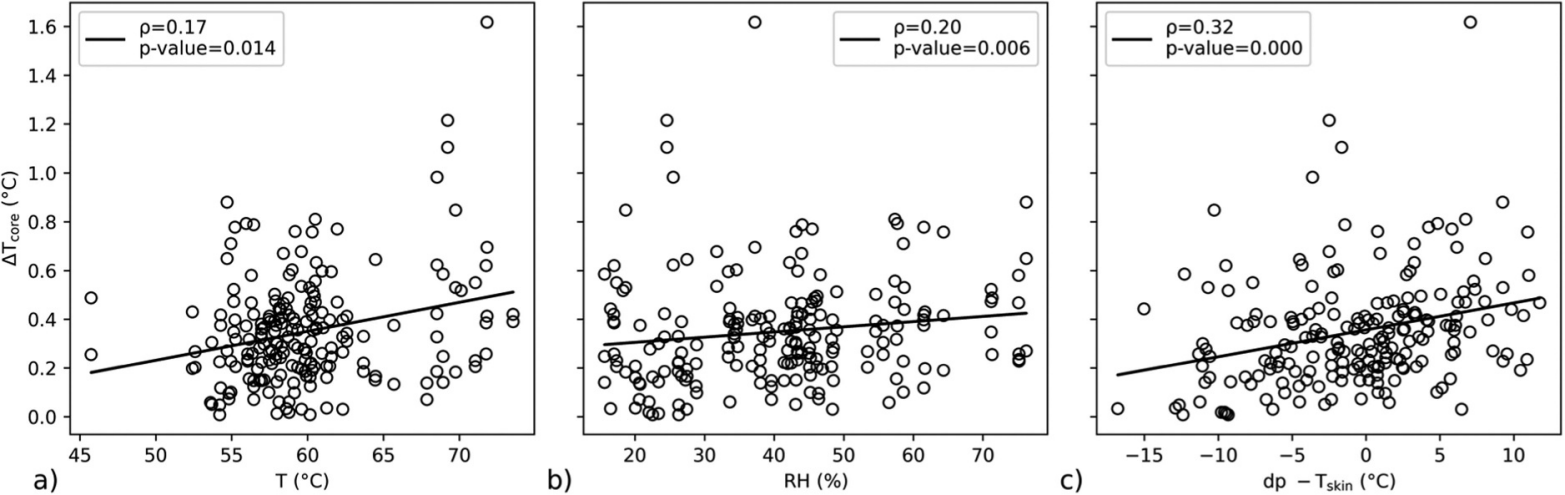
Correlation between T (a), RH (b), or dp-T_skin_ (c) and ∆T_core_: scatter plots with Spearman’s rho (ρ) and *p*-value.

Figure 4 illustrates the correlations between T, RH, or dp‑T_skin_, and ∆T_core_, which were all weak but statistically significant.

### Change in body mass

Mean change in body mass was −0.10 kg ±0.07 kg. Sauna temperature was negatively associated with the change in body mass, i.e. a variable describing the amount of sweating (β = −0.0025 kg, 95% CI: −0.047 to −0.0002, *p* = 0.031). In addition, the 3^rd^ and 4^th^ sauna sessions were statistically significantly associated with ∆m (β = −0.021 kg, 95% CI: −0.039 to −0.0021, *p* = 0.029 and β = −0.027 kg, 95% CI: −0.050 to −0.0034, *p* = 0.025). When sex, physical activity, body fat percentage and skin area were added to the model, the 3^rd^ and 4^th^ sauna sessions still had negative associations with ∆m. In addition, physical activity and skin area were associated with ∆m (β = −0.0056 kg, 95% CI: −0.011 to −0.0007, *p* = 0.025 and β = −0.096 kg, 95% CI: −0.17 to −0.021, *p* = 0.012).

In a GEE model with dp-T_skin_ and sauna session, only the 3^rd^ and 4^th^ sauna session were negatively associated with ∆m. When sex, physical activity, body fat percentage and skin area were added in the model, physical activity had also a negative association with ∆m (β = −0.0050 kg, 95% CI: −0.0097 to −0.0003, *p* = 0.039) and 3^rd^ and 4^th^ sauna sessions still had their statistically significant effect.

**Figure 5.**
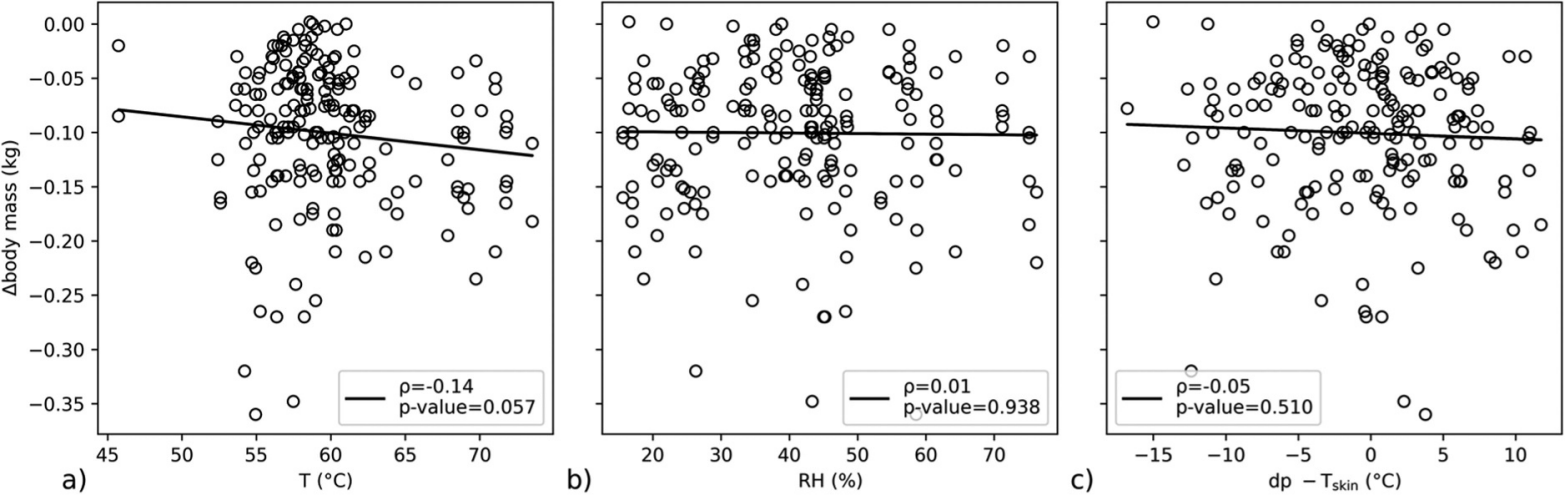
Correlation between T (a), RH (b), or dp-T_skin_ (c) and ∆body mass: scatter plots with Spearman’s rho (ρ) and *p*-value.

Figure 5 illustrates the correlations between T, RH, or dp‑T_skin_, and ∆body mass, which were not statistically significant.

### Perceptual measures

Perceptual responses were primarily driven by temperature, with smaller contributions from humidity. In a GEE model including sauna session, sauna temperature and relative humidity, T, and RH showed positive associations with thermal sensation (β = 0.081, 95% CI: 0.046–0.11, *p* = 0.000 and β = 0.013, 95% CI: 0.0017–0.024, *p* = 0.024). Additionally, the 2nd sauna session was positively associated with thermal sensation. When sex, physical activity, body fat percentage, and skin area were included in the model, T (β = 0.078, 95% CI: 0.043–0.11, *p* = 0.000) and 2^nd^ sauna session remained positively associated with thermal sensation, and physical activity also showed a positive association (β = 0.084, 95% CI: 0.0096–0.16, *p* = 0.027).

In a GEE model with sauna sessions, T and RH, 2^nd^, 3^rd^ and 4^th^ sessions were positively associated with thermal comfort (see supplementary material). The results were very similar when sex, physical activity, body fat percentage and skin area were included in the model. Furthermore, results were similar for sauna session and dp-T_skin_: sauna sessions were positively associated with thermal comfort with and without sex, physical activity, body fat percentage and skin area in the model.

## Discussion

This study examined the effects of sauna temperature and relative humidity on cardiovascular and thermoregulatory responses, as well as perceived thermal sensation and comfort. The main findings were that (i) both temperature and humidity significantly influenced physiological responses, (ii) heart rate increased substantially during sauna exposure while remaining consistent across repeated sessions, and (iii) increases in core temperature were moderate and showed only minor differences between sessions. Importantly, the results highlight the humidity as a meaningful contributor to thermal load in sauna environments. It is reasonable to assume that higher temperatures elicit greater physiological responses. Similarly, humid conditions are generally known to impose greater physiological strain on the cardiovascular system than dry conditions at the same temperature [20]. However, less is known about the specific role of humidity in the context of sauna bathing.

In this study, heart rate and core temperature increased during the 10-minute sauna sessions. Increases in HR and T_core_ were influenced by both the temperature and relative humidity of the sauna: each 1°C increase resulted in a 1.42 bpm greater change in heart rate, and each 1%-point increase in relative humidity resulted in a 0.38 bpm greater change in heart rate. On average, the heart rate increased by 41.6 bpm during a single sauna session, which is a relatively large change in only 10 minutes. It is noteworthy that protocols, including the timing of HR measurement, and study participant characteristics, vary considerably across studies. Leach et al. [9] reported an 18-bpm change in young participants and a 15-bpm change in middle-aged participants when HR was measured immediately before and after the sauna exposure (two 20-minute sessions in a dry 80°C sauna with a 10-minute rest interval). Ketelhut & Ketelhut [5] detected a mean change of 53.5 bpm in HR after 25 minutes in a dry and hot sauna in healthy adults, when HR at the end of the sauna exposure was compared to resting HR. In a study by Laukkanen et al. [21], participants with one or more cardiovascular risk factors (mean age 52 ± 8.8 years) experienced a mean increase of 43 bpm in heart rate after two 15-minute sauna sessions at 73°C.

In the present study, ∆T_core_ was on average 0.4°C during a single 10-minute sauna session, consistent with Atencio et al. [22], who reported a 0.4°C increase in core temperature after three 10-minute sauna sessions at 80°C (15%–20% humidity) with 5-minute rests between the sessions. These changes are smaller than those reported by Leach et al. [9], who found a 1.5°C average increase after two 20-minute sauna sessions in an 80°C dry sauna and Heinonen et al. [23] who reported tympanic temperature rising by 2°C after two 15 minutes sauna sessions (73°C). In our study, both temperature and humidity appeared to be associated with an increase in core temperature. A one-degree increase in sauna temperature resulted in a 0.032°C greater ∆T_core_, while a one-percentage-point increase in relative humidity led to a 0.0076°C greater ∆T_core_.

In traditional Finnish sauna use, water is frequently thrown onto heated stones (*löyly*), causing rapid and transient increases in humidity. This dynamic fluctuation in humidity is rarely captured in controlled experimental studies, which often describe conditions as “dry.” Consequently, previous studies may underestimate the physiological strain associated with real-world sauna use. Although some studies have attempted to incorporate steam exposure, the temporal dynamics and magnitude of humidity changes remain poorly characterized. Atencio et al. [22] reported that two ladles of water were thrown onto the sauna heater at the beginning of each 10‑minute sauna session. However, the mean relative humidity was only 17%, although temporarily the relative humidity increased to 40%. Pilch et al. [6], compared the physiological reactions to hot dry (91°C, 5–18%) and steam sauna (59°C, 60.5% humidity) exposures and concluded that wet sauna places greater physiological stress than dry sauna due to high humidity reducing thermoregulation mechanisms.

Accordingly, high humidity in sauna impairs the body’s primary cooling mechanism by preventing sweat from evaporating effectively, which induces significantly greater cardiovascular strain compared to dry heat. This increased physiological stress causes the heart rate to rise more substantially as the cardiovascular system works harder to redistribute blood to the skin in an attempt to maintain thermal homeostasis [24].

In this study, the significance of humidity was assessed using the difference between dew point and skin temperature as a variable representing water vapor condensation on the skin. When skin temperature is below the dew point, water vapor in the air condenses as liquid water on the skin surface. The association between dp – T_skin_ and ΔHR was weak and not consistently observed across models, suggesting that although condensation may contribute mechanistically, its measurable impact under these conditions is limited. In contrast, humidity alone showed a clearer association with physiological responses, indicating that the overall moisture content of the sauna air may be more influential than condensation itself. However, the rapid increase in humidity caused by löyly throwing likely alters both the thermal and moisture environment simultaneously, making it difficult to fully separate the independent effects of humidity and condensation. The effects of löyly throwing and the associated humidity-induced condensation on physiological variables, as well as the perceived comfort of sauna bathing, should therefore be examined in greater detail in future studies.

Sweating was estimated from changes in body mass. On average, the changes in body mass were small (mean −0.100 kg), and subject to measurement error. For comparison, Gravel et al. [8] reported a 0.21 kg weight loss after 10-minute 80°C sauna exposure, Atencio et al. [22] a 0.66 kg weight loss after 3 × 10-minute sauna bathing. Factors such as moisture retained in swimwear, and water accumulation in hair may have affected the accuracy of these estimates. In addition, fluid intake during breaks may have influenced fluid balance and, consequently, the amount of sweating. Body mass measurement was also prone to other errors (e.g. participant movement, unintended fluid intake) and due to these methodological challenges, some measurement results had to be excluded from the analysis.

Perceptual responses followed a similar pattern to physiological responses, with temperature as the primary determinant of thermal sensation and humidity having a smaller but detectable effect. Interestingly, repeated sessions were associated with increased thermal comfort, which may reflect habituation or increased tolerance to heat exposure. In a previous study, Pilch et al. [6] found that subjective heat comfort is greater in more humid and lower temperature sauna bath.

In studies of the traditional sauna, temperature has often been 73–95°C [5,8,10,25,6,22,23] and measured at a single height, with the conditions reported as dry. However, in the traditional Finnish sauna, water is customarily poured onto the stones, resulting in conditions that vary substantially depending on the amount of steam generated. Therefore, single-point measurements may not adequately represent the overall thermal exposure experienced by an individual. A major strength of the present study was the detailed assessment of environmental conditions, with temperature and humidity continuously measured at multiple spatial locations within the sauna (two heights and three horizontal positions). Compared with previous studies, measured temperatures were lower and humidity levels higher than typically reported, likely reflecting differences between thermostat settings and actual environmental conditions, as well as spatial variation within the sauna room. Similar discrepancies have also been reported previously; for example, Atencio et al. [22] measured a temperature of 66°C (17% humidity) at the top of the sauna despite a thermostat setting of 80°C, while Lee et al. [10] reported a thermostat setting of 84°C but a measured mean internal temperature of 73 ± 2°C. These findings highlight the importance of accurate and spatially comprehensive environmental measurements when interpreting and comparing studies on sauna exposure.

This study has several limitations. The repeated-session design may have introduced subtle carry-over effects despite the recovery periods, as the same participants completed all four sauna sessions with 30-minute rest intervals between exposures. However, no clear cumulative responses were observed. Although heart rate can remain elevated after sauna exposure [5,9], Lee et al. [10] reported a return to baseline within 30 minutes. In the present study, subsequent sauna sessions did not differ from the first session in ∆HR, and only the third session showed a statistically significant difference in ∆T_core_ compared to the first session (−0.067°C), which was physiologically trivial. Sauna exposure time was also relatively short, as each 10-minute session was treated as a separate exposure and recovery periods were longer than in several previous studies involving repeated sauna exposure [6,8,9,22]. In addition, considerable inter-individual variability may have influenced the results, highlighting the importance of accounting for differences in thermal sensitivity. Since interaction effects involving the covariates and potential sex differences were not examined, the applicability of these findings to particular subgroups may be limited.

Furthermore, as caffeine consumption was not controlled and participants’ medication use was not considered in the analyses, some of the findings may be biased due to uncontrolled factors. In future studies, physical fitness should also be measured objectively rather than relying on participants’ self-reported physical activity, and monitoring blood pressure would provide additional insight into cardiovascular responses. Last, most participants in this study were habitual sauna users, which may have influenced their perception of thermal sensation and comfort. Regular exposure to heat may increase tolerance, allowing conditions to be perceived as hot but comfortable. This may partly explain the discrepancy between objectively measured physiological strain and subjective perceptions of comfort, although physiological responses to heat exposure may also be attenuated following regular heat exposure [26].

## Conclusion

In conclusion, both temperature and humidity significantly influence physiological responses to sauna exposure. While temperature is the dominant factor, humidity independently increases cardiovascular and thermoregulatory strain by impairing evaporative heat loss. These findings highlight the importance of considering humidity in both experimental and real-world sauna settings. Although the present study was designed to reflect ecologically valid sauna bathing practices, future research using more controlled experimental conditions is warranted to further isolate the independent effects of temperature and humidity. Given the substantial inter-individual variability observed, monitoring physiological parameters and optimizing environmental conditions (including humidity) may help to enhance the safety of sauna bathing and optimize its responses and potential health benefits in future applications.

## Supplementary Material

Supplementary_material.xlsx

Supplementary_table2.docx

## Data Availability

Data will be made available on reasonable request.

## Abbreviations

bpm: beats per minute
dp: dew point
GEE: generalized estimating equations model
HR: heart rate
m: body mass
PBF: percent of body fat
RH: relative humidity
SD: standard deviation
T: temperature
T_core_: core temperature
T_skin_: skin temperature
