# Supplementary material for: Temperature and humidity independently influence thermoregulatory responses during Finnish sauna bathing
Source: Temperature (Austin). 2026 Jul 11;13(3):289–99. doi: 10.1080/23328940.2026.2698162 (PMC13540098; doi:10.1080/23328940.2026.2698162)
Supplement: Supplementary file 1 [file KTMP_A_2698162_SM6582.docx]

|  | N | Physical activity (0-7) | Age (years) | Height (cm) | Weight (kg) | PBF* (%) |
| --- | --- | --- | --- | --- | --- | --- |
| All | 19 | 4.6 (1.5) | 38.1 (11.5) | 177 (8.9) | 85 (19) | 24 (8.5) |
| Female | 6 | 5 (1.7) | 35.8 (10.4) | 170 (6.7) | 69 (13) | 28 (8.4) |
| Male | 13 | 4.4 (1.4) | 39.1 (12.3) | 180 (7.9) | 93 (17) | 23 (8.3) |

Supplementary table 2. Details from excluded study participants.
